# Visceral Leishmaniasis with Endobronchial Involvement in an Immunocompetent Adult

**DOI:** 10.1155/2011/561985

**Published:** 2011-03-10

**Authors:** Konstantinos Kotsifas, Eugenios Metaxas, Ioannis Koutsouvelis, Athanassios Skoutelis, Panayiota Kara, George Tatsis

**Affiliations:** ^1^Pulmonary Medicine Department, Evaggelismos General Hospital, 45-47 Ypsilantou Street, 10676 Athens, Greece; ^2^5th Department of Internal Medicine, Evaggelismos General Hospital, Greece; ^3^Department of Pathology, Evaggelismos General Hospital, Athens, Greece

## Abstract

Visceral leishmaniasis is characterized by fever, cachexia, hepatosplenomegaly, pancytopenia, and hypergammaglobulinemia. Cough may be a presenting symptom as well. However, pulmonary involvement is considered rare and mainly described in immunocompromised patients. We describe a case of an immunocompetent adult whose clinical presentation was dominated by cough and hemoptysis. Bronchoscopy revealed a discreet polypoid mucosal endobronchial lesion whose biopsy yielded Leishmania amastigotes within histiocytes. Transbronchial needle biopsy of a right paratracheal lymph node was also positive. Leishmania amastigotes were also found on bone marrow and liver biopsies. Treatment with IV Amphotericin B was successful. In conclusion, cough should not be overlooked as a presenting symptom of visceral leishmaniasis and may be a sign of pulmonary involvement.

## 1. Introduction

Leishmaniasis is a parasitic disease with a wide range of clinical manifestations depending on the interaction between different *Leishmania* species and immune host responses.

Most infections being asymptomatic, three main clinical syndromes are recognized, namely, visceral, cutaneous, and mucocutaneous. 

Visceral leishmaniasis (VL) is the most severe form with an annual incidence of about 500 000 cases and a respective mortality of 80 000 [1].

With 90% of cases occurring in five countries, namely Bangladesh, NE India, Nepal, Sudan, and NE Brazil, the disease could be mistakenly considered rare in Europe [2].

Nevertheless, the Mediterranean littoral constitutes an endemic area, with *Leishmania infantum* as the causative agent. Factors such as immigration and global warming may further contribute to disease propagation [3, 4]. 

Moreover, the increase in the number of immunocompromised patients has resulted in an increased incidence of VL and novel clinical presentations [5]. 

VL is characterized by fever, cachexia, hepatosplenomegaly (predominantly splenomegaly), pancytopenia, and hypergammaglobulinemia.

Lung involvement is considered rare, especially in immunocompetent patients. 

We report a case of VL in an immunocompetent man with clinical and bronchoscopically confirmed endobronchial and mediastinal involvement and briefly review the pertinent literature.

## 2. Case Presentation

A 40-year-old man of Albanian descent, living in Athens the last 20 years, was admitted on February 2008. His symptoms began one year prior to admission with dry cough and gradual weight loss of a total of 30 kg. Night sweats and scant hemoptysis appeared the last month. A limited workup 6 months ago had revealed the presence of chronic active hepatitis B. 

The patient was working in a restaurant, was a nonsmoker, and had no other remarkable medical history. Clinical examination revealed cachexia, hepatosplenomegaly, and small, discrete, and nontender axillary and cervical lymph nodes. There were no clinical signs of liver failure or portal hypertension such as jaundice, palmar erythema, spider nevi, encephalopathy, or ascites. 

Laboratory tests showed pancytopenia, elevated hepatic aminotransferases (3-4 times the upper limit) and polyclonal hypergammaglobulinemia. Erythrocyte Sedimentation Rate was 100 mm/h. Serum albumin, bilirubin and prothrombin time, were within normal limits. HIV was tested negative by ELISA twice by three months apart. Antibodies against Hepatitis C virus were negative as well. An abdominal ultrasound revealed increased dimensions of the liver and spleen and the presence of enlarged lymph nodes at the hepatic hilum. Diameter and flow through the portal vein were normal, and no ascites was detected. A Computerized Tomography scan of the chest revealed limited adenopathy; a 2 × 1 cm mass at the right hilum, and a 1.2 cm right lower paratracheal lymph node with hypodense centre. 

**Figure 1:**
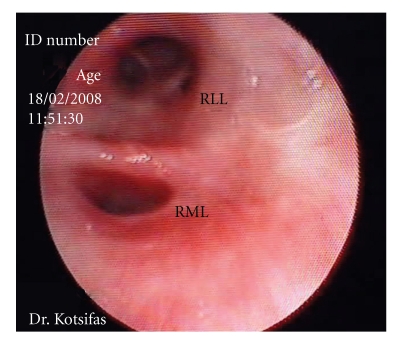
Endoscopic view from the lower part of the bronchus intermedius. The mucosa appears inflamed and slightly nodular at the level of the middle lobe bronchus, on the anteromedial wall. On the middle lobe carina, there is a discreet polypoid lesion. RML: right middle lobe bronchus. RLL: right lower lobe bronchus.

**Figure 2:**
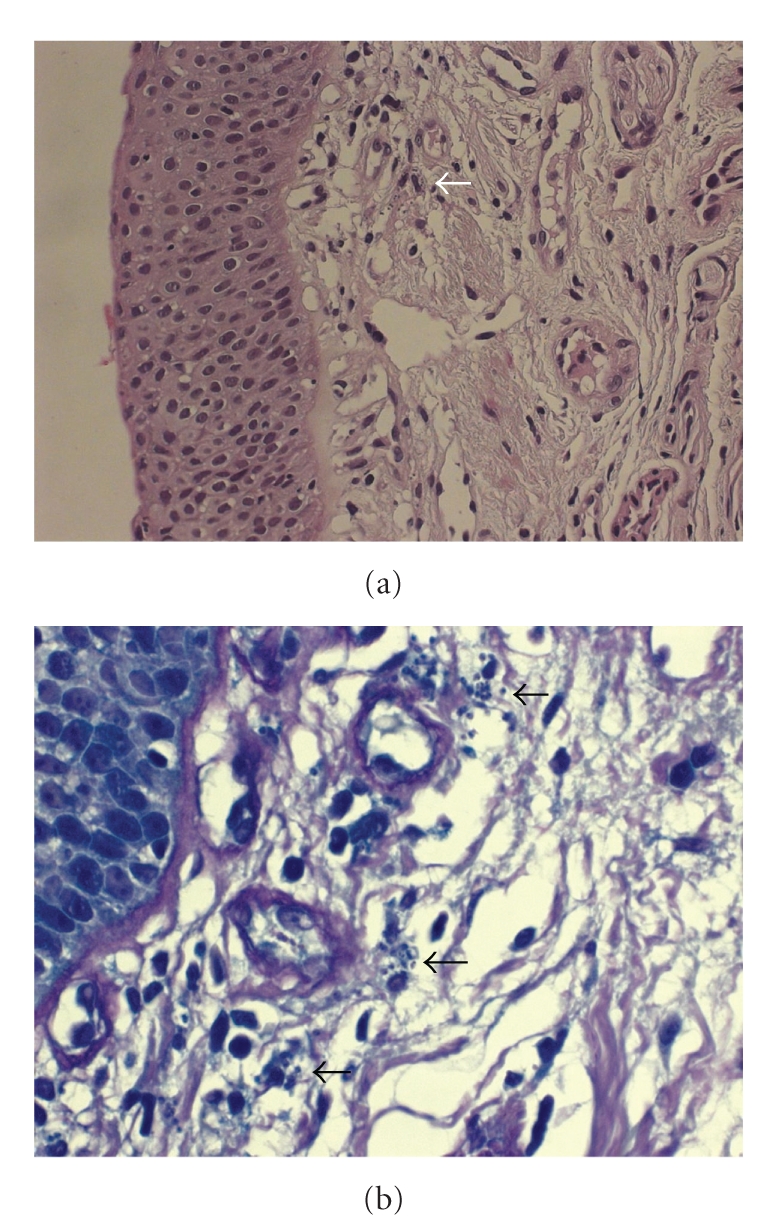
(a) Bronchial biopsy showing epithelial hyperplasia and few Leishmania amastigotes within subepithelial histiocytes (white arrowhead). (Hematoxylin-Eosin ×200). (b) A higher magnification view of the positive area. Several collections of Leishmania amastigotes are indicated by black arrowheads (Giemsa ×1000).

**Figure 3:**
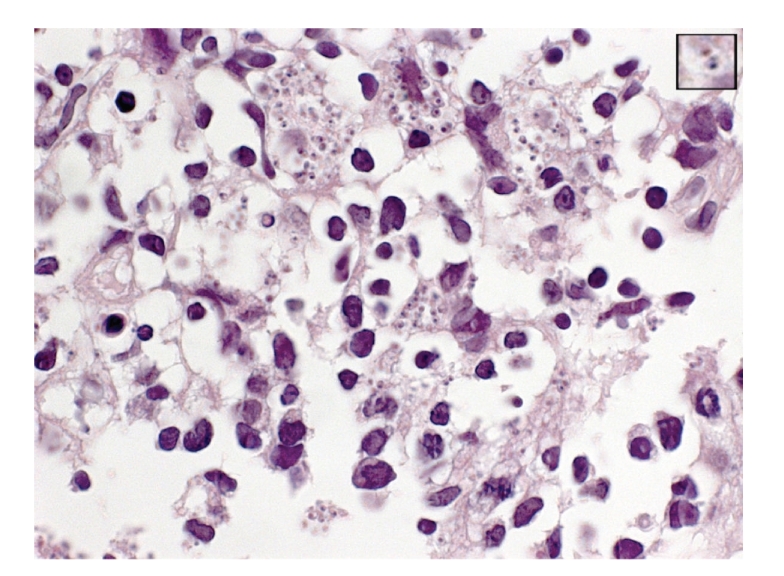
Transbronchial needle biopsy of a mediastinal lymph node showing histiocytes containing abundant Leishmania amastigotes (H-E × 1000). Insert shows a close-up view of an amastigote. Its ovoid shape, eccentric nucleus, and kinetoplast are discerned.

On bronchoscopy, epistaxis and pharyngeal inflammation consistent with candidiasis were noticed. There was mild diffuse redness throughout the tracheobronchial tree. Inflammation was more prominent on the medial aspect of the bronchus intermedius extending into the middle lobe bronchus. Interestingly, a discreet mucosal polypoid lesion was observed on the middle lobe carina (Figure 1). Bronchial biopsies taken from this lesion revealed epithelial hyperplasia and several Leishmania amastigotes within histiocytes (Figures 2(a) and 2(b)). Transbronchial needle biopsies (TBNB) targeted at the right lower paratracheal lymph node also revealed histiocytes containing abundant Leishmania amastigotes (Figure 3).

A bone marrow biopsy was also positive for Leishmania amastigotes, as was a liver biopsy. In the latter, there was considerable hepatic inflammation, attributed to both Leishmania and HBV infection, along with minimal fibrosis but not cirrhosis. Fluorescent antibodies against Leishmania were positive in high titers of ≥1/640.

The patient was treated with IV liposomal Amphotericin B with prompt clinical and laboratory improvement. Six months later, white blood cell count and differential, lymphocyte subpopulations, and immunoglobulin levels were within normal limits. Due to the persistence of increased hepatic aminotransferases (1.5 times the upper limit) the patient began treatment with telbivudine. On the first and second year follow up, the patient remained in excellent clinical condition and had normal laboratory values.

## 3. Discussion

We describe a case of visceral leishmaniasis in an immunocompetent adult who presented with cough and hemoptysis along with the typical clinical features of the disease. Involvement of bronchial mucosa resulted in mild diffuse inflammation, but most importantly in a visible endobronchial lesion whose biopsy yielded amastigotes of Leishmania within histiocytes. Histology was also positive for a lower paratracheal node accessed through transbronchial needle biopsy.

Clinical lung involvement in VL is considered rare. However, a persistent dry cough is an overlooked, yet quite common feature of VL, observed in up to 83% of affected patients [6–8]. It may be a presenting symptom and may accompany the illness until effective treatment is administered. While cough can originate outside the lungs, pulmonary involvement in VL has been indeed demonstrated. 

Interstitial pneumonitis with a mainly mononuclear cell infiltrate and foci of fibrosis has been shown in experimentally infected mice and naturally infected dogs [9, 10]. Furthermore, following earlier observations in humans, an autopsy study in Brazil demonstrated interstitial pneumonitis and Leishmania antigenic material in 10 out of 13 cases of VL. The parasite itself was identified in 3 cases. Lesions within the bronchi were not detected. Clinical features were unfortunately not recorded [11].

A Th2 polarized immune response with deficient IFN-gamma production has been recently demonstrated within the lung interstitium in cases of VL with pulmonary involvement [12]. This inflammatory pattern—which is consistent with the immune response in other affected organs—could be responsible for the increased susceptibility of patients with VL to respiratory infections. 

Bronchial involvement in VL has been reported in an immunocompetent patient from Sudan; *Leishmania donovani* was identified using PCR in bronchoalveolar lavage and bronchial biopsies. This man had also involvement of the nasal and oropharyngeal mucosa as well as massive mediastinal lymphadenopathy and generalized inflammation of the bronchial tree. No discrete lesion was identified on the bronchial mucosa, and amastigotes were not seen on stains. Histopathology was characterized by granulomatous inflammation. Moreover, the authors concluded that this patient had an immunological profile more akin to mucocutaneous or post kala azar dermal leishmaniasis than to VL [13]. 

Another case of endobronchial involvement in VL has been described in a man with significant immunosuppression and HIV-related infections. Bronchial lesions were reported to macroscopically resemble Kaposi sarcoma. Leishmania amastigotes were found within submucosal histiocytes as in our case but without concomitant epithelial reaction. Bronchoscopic images were not provided [14].

Predictably, pulmonary involvement in VL is more often reported in immunocompromised patients, particularly those infected by HIV. In fact, Leishmania has emerged as an important opportunistic infection in the HIV-positive population; in southern Europe, 25% to 70% of adult VL cases involve HIV-positive patients [2]. It is worth noting that Leishmania infection may by itself foster HIV infection by promoting viral replication [15]. 

VL in the presence of HIV may present atypical clinical manifestations and affect unusual sites [5]. Leishmania was first described to affect the lungs of a patient with AIDS in 1991 [16]. Subsequently, it has been described as a cause of pulmonary infiltrates [17], a solitary pulmonary nodule [18], diffuse pulmonary and pleural disease [19, 20], lung masses [21], and bronchioloalveolar adenoma [22].

Apart from HIV infection, VL is diagnosed with increased frequency among other immunocompromised groups such as solid organ transplant recipients, including of lung transplants [23].

Our patient's history and clinical course after treating VL did not suggest any concrete immune defect. Hepatitis B virus is not known to directly affect the immune system; still, cirrhosis and liver failure may be associated with some degree of immune suppression, mainly, in advanced stages. However, most of the hepatic dysfunction in our patient should be attributed to Leishmania infection as shown by the clinical and laboratory recovery under treatment. Hepatic failure was never present and liver histopathology findings were not sufficient to document cirrhosis. In fact, hepatic inflammation and fibrosis, occasionally leading to cirrhosis, have been recognized as complications of the hepatic involvement in VL [24]. Nevertheless, we cannot definitely exclude a possible contribution of subtle liver failure caused by chronic hepatitis B on the acquisition and expression of Leishmania infection in our patient. On the other hand, it is also possible that immune dysregulation caused by Leishmania infection [25] might have promoted and aggravated HBV infection [26].

Clinical and laboratory features of VL overlap with those of chronic hepatitis B with cirrhosis [27]. Indeed, in our patient, enlargement of the liver and spleen together with laboratory abnormalities was initially attributed to chronic HBV infection. The presence of cough and particularly hemoptysis further confounded the diagnosis. Bleeding from the tracheobronchial tree was not observed during bronchoscopy, and consequently, an upper airway origin could be equally possible. In light of the final diagnosis, epistaxis and pharyngeal inflammation could represent Leishmania infection of the upper airway mucosa; no biopsies were taken to verify this hypothesis. It is noteworthy that the bronchoscopic appearance of the middle lobe carina lesion was similar to lesions described on the larynx [28, 29]. 

Mucocutaneous disease is generally reported in Latin America and is caused by *Leishmania braziliensis* [1]. However, mucosal involvement of the oral cavity—including tongue and tonsils—the nose, the pharynx, and the larynx, is an infrequent but well-described complication of *Leishmania infantum* infection. It is not confined to immunocompromised patients and may occur either isolated or in the context of visceral disease. Lesions may appear as nodular swelling or tumor like and less often as ulcerative. A chronic inflammatory infiltrate is observed on histopathology. Granulomatous inflammation is often but not universally demonstrated [29].

Histopathology of the endobronchial lesion was characterized by the absence of significant submucosal inflammation despite the presence of infected histiocytes, and by the hyperplasia of the respiratory epithelium. Cases of documented endobronchial leishmaniasis are rare, and therefore we cannot draw meaningful conclusions from these observations. Nevertheless, these findings are not unprecedented since both insignificant inflammation [14] and hyperplastic epithelial cells [21] have been reported in respective cases. More importantly, a significant hyperplasia of the respiratory epithelium of terminal and respiratory bronchioles has been observed in dogs naturally infected with *Leishmania chagasi* [10].

Despite the limited number of reported cases, the diversity of clinical and histopathologic expressions of pulmonary involvement in VL is remarkable. The interactions between different Leishmania species and immune host responses that account for these differences are not yet elucidated. 

In this context, it would have been interesting to characterize the species of the parasite that affected our patient. Unfortunately, neither culture nor PCR for Leishmania species are available in our hospital. In Greece, VL is caused by *Leishmania infantum*, zymodeme MON-1. Phlebotomine sand flies are responsible for transmission, and dogs are the only known reservoir hosts. Rare cases of cutaneous disease are caused by the same parasite. Seroprevalence in northern Greece has been recently assessed between 2–2.8% [30] which is close to that of other south European countries. Annual incidence in Greece is estimated between 0.19 and 1.0 cases per 100 000 inhabitants [6, 30, 31].

## 4. Conclusion

We describe a unique case of a discrete endobronchial lesion related to Leishmania infection. It is possible that the application of diagnostic methods not available in the past, such as fiberoptic bronchoscopy and TBNB, will enrich our knowledge on the pulmonary involvement in VL. Meanwhile, Leishmania infection may increase its prevalence, propagate geographically, and present novel clinical features in relation to immigration, climate change and immunosuppression. 

Cough should not be overlooked as a presenting symptom of visceral leishmaniasis and may be a sign of pulmonary involvement.
